# The Eleventh Annual Meeting of the Indiana State Dental Association

**Published:** 1869-07

**Authors:** S. B. Brown


### THE ELEVENTH ANNUAL MEETING OF THE INDIANA STATE DENTAL ASSOCIATION.

Tuesday, June 29, 1869.

Met at the rooms of the Young Men's Christian Association at 2 o'clock P. M.

President S. M. Good being absent, First Vice President W. C. Stanley called the meeting to order.

Members present—W. C. Stanley, C. C. Burgess, E. M. Morrison, A. T. Keightley, W. F. Morrill, Thomas H. Martin, H. H. Morrison, J. B. Harlan, P. G. C. Hunt, W. H. Pifer, A. O. Rawls, Merit Wells, P. F. Hancock, J. M. Shaw, L. W. Munhall, J. F. Johnston, G. A. Wells.

Minutes of last meeting read and approved.

Board of Censors—Drs. Morrill, Johnston and Keightley.

The names of Drs. L. H. Bartholomew, of Terre Haute, and S. B. Brown, of Fort Wayne, were presented for membership and both elected.

On motion it was agreed to go into election of officers, which resulted as follows:

President—Dr. John F. Johnston, Indianapolis.

First Vice President—Dr. A. M. Moore, Lafayette.

Second Vice President—Dr. W. H. Pifer, Lafayette.

Secretary—Dr. Seneca B. Brown, Fort Wayne.

Treasurer—Dr. C. C. Burgess, Indianapolis.

Drs. Wm. E. Dunn and Will Taft, of Ohio, being present, were elected honorary members.

Dr. A. M. Moore read an essay on the "Physiological Effects of Nitrous Oxide Gas."

The thanks of the Association were tendered for his able paper, and a copy was asked for publication.

Dr. John F. Johnston followed with an essay on "Sustaining the Code of Ethics," which met the hearty approval of the Association by a vote of thanks and a copy requested for publication.

On motion a committee, consisting of Drs. Moore, Morrill and Keightley, were appointed to ascertain if there had been any violation of the Code of Ethics by any member of the Association.

On motion the Committee on Procuring Passage of Dental Bill in Legislature were continued, after Dr. Hunts report on same, the committee consisting of Drs. Hunt, Moore, Johnston, Keightley and Richardson.

The following subjects having been selected for discussion, were now taken up:

1. The best method of controlling the oral secretions in Dental operations.
2. The best protection for exposed nerves.
3. What are the indications for extracting the teeth?
4. Dental Therapeutics.
5. Mechanical Dentistry.
6. Miscellaneous.

The first subject, "The best method of controlling the oral secretions in Dental operations" was now taken up. Dr. Will Taft regards the rubber dam as superior to all other methods, and interested the Association by showing the manner of application.

Drs. Morrill and Moore followed in the discussion.

Moved to adjourn till a quarter before 8 o'clock.

### EVENING SESSION.

The discussion on the first subject being continued, Drs. Stanley, Rawls, Dunn and Hancock participated, when the subject was disposed of and the second subject taken up—"The best protection for exposed nerves."

Dr. A. O. Rawls read a very able paper on the subject, which received a vote of thanks and a copy requested for publication.

Drs. Morrill, Moore and Dunn followed.

On motion adjourned to meet to-morrow at 8 o'clock, at the Dental Depot of Messrs. Strong & Smith, for clinical operations.

### SECOND DAY—MORNING SESSION.

Association met, pursuant to adjournment, at the rooms of Strong & Smith, for clinics.

Dr. Will Taft demonstrated to the members present, his method of applying rubber dam to the necks of teeth, by means of waxed floss silk; then removing the silk the dam remains in place without the aid of ligatures, wedges, &c. An hour having been spent here with much interest and profit, the members proceeded to their regular place of meeting.

Association called to order by President Johnston. Minutes of preceding meeting read and adopted.

On motion, Dr. N. W. Williams, of Xenia, Ohio, being present, was elected to honorary membership.

The committee appointed to ascertain if there had been any violation of the Code of Ethics by members of this Association, presented the following report:

"Your committee, to whom were referred the inquiries into infractions of the Dental Code, would most respectfully report that several cases have been before your committee. The names of them are Drs. Chappell, of Knightstown, Purcell, of Indianapolis, Munhall, of Indianapolis, Morrison, of Greencastle, who have either designedly or inadvertently violated that clause in the Code in respect to advertising to do work at cheap rates, and other particulars which we do not deem necessary to mention. Your committee would respectfully recommend that these gentlemen be cited to appear before them and show cause why they should not be expelled from the Society for these violations."

Drs. Morrison and Munhall having made explanations and promised a strict adherence to the Code in future, they were continued as worthy members of the Association.

Drs. Chappell and Purcell did not appear, and as there was abundant evidence to support the charges brought against them, they were, by vote of the Association, expelled.

The Committee on Inquiry into Infractions of Dental Ethics were continued.

On motion O. F. Britton, of Champaign, Illinois, was made an honorary member.

Dr. W. E. Driscoll, of Bedford, Indiana, was, on ballot, elected a member of this Association.

The regular order of business being suspended, Dr. Dunn, of Dayton, Ohio, exhibited cases of his porcelain work.

Dr. W. F. Morrill read a paper, the subject of which was "How to conduct an office practice." This was a production of much merit and well calculated to elevate professional bearing. We understand it will be published in pamphlet form.

The hearty thanks of the Association were expressed by vote, and a copy solicited for publication.

On motion adjourned to 2 o'clock P. M.

AFTERNOON SESSION.

Association called to order by President Johnston at the hour appointed.

The retiring President, Dr. Good, being absent, a letter from him was read by the President, which elicited a vote of thanks and ordered on file.

By consent the second subject for discussion was concluded and the third subject, "What are the indications for extracting the teeth," taken up.

Dr. W. C. Stanley read a paper on the above subject, which was ordered to be put on file.

Discussions followed by Drs. Rawls, Wells and Williams.

The fourth subject, on "Dental Therapeutics," was next in order, and interesting accounts of practice given by Drs. Williams, Morrill, Hunt and Rawls, when the subject was passed and "Mechanical Dentistry," the next and fifth subject, taken up. Dr. Dunn represented the porcelain style of work and claimed its superiority over other styles, excepting continuous gum.

Dr. W. Taft was called upon to give some account of rose pearl, which he did, saying he did not wish to be understood as an especial advocate of that style, but had no doubt of its advantage over rubber.

Dr. Keightley presented views favorable to the aluminum base.

On motion the Association proceeded to the next and last subject for discussion—"Miscellaneous."

Dr. Moore presented resolutions, viz.:

*Resolved*, That the election of officers hereafter shall be held after the consideration of the regular subjects have been disposed of.

Also, that the President shall appoint a member to open discussions on each subject.

Carried.

Dr. Williams, of Xenia, Ohio, spoke of the kindness extended to the visitors from his own State, and extended to the members of this Association a most cordial invitation to attend the Association of their State at Columbus.

On motion adjourned to meet at 8 o'clock P. M.

EVENING SESSION.

President Johnston in the chair.

An Auditing Committee, consisting of Drs. Lea W. Munhall and Seneca B. Brown, of Fort Wayne, was appointed, and after examining the Treasurer's accounts, submitted the following report:

| | |
|---|---|
| Amount of cash on hand for last year's report | $95 28 |
| Received from initiation fees and dues | 125 00 |
| Total | $220 28 |
| Amount paid for incidental expenses | 117 89 |
| Leaving balance in the Treasury | $102 39 |

The report was received and committee discharged.

The Executive Committee for the succeeding year was announced as follows, by the chair: Dr. W. C. Stanley, of Dublin; Dr. A. O. Rawls, of Connersville; Dr. W. L. Heiskell, of Indianapolis.

Dr. Davis, of Cincinnati, exhibited the Hall's Gas Burner, which was very highly spoken of by nearly all who had used it.

A paper on "Microscopy of the Dentinal Tubuli," by S. P. Cutler, M. D., A. E. G., D. D. S., of Holly Springs, Mississippi, was read, and a motion that it be published and that the Secretary be directed to communicate to the author a vote of thanks; prevailed.

No delegates were appointed to the American Dental Association. The President and Secretary were authorized to issue certificates to any member who may desire to attend, on application.

At 10 o'clock P. M. the Association adjourned, to meet in the city of Indianapolis, on the last Tuesday of June, 1870.

S. B. Brown, *Secretary.*

